# Splicing factor SF3B1^K700E^ mutant dysregulates erythroid differentiation via aberrant alternative splicing of transcription factor TAL1

**DOI:** 10.1371/journal.pone.0175523

**Published:** 2017-05-18

**Authors:** Shuiling Jin, Hairui Su, Ngoc-Tung Tran, Jing Song, Sydney S. Lu, Ying Li, Suming Huang, Omar Abdel-Wahab, Yanyan Liu, Xinyang Zhao

**Affiliations:** 1 Department of Internal Medicine, Henan Cancer Hospital & Affiliated Cancer Hospital of Zhengzhou University, Zhengzhou, China; 2 Department of Biochemistry and Molecular Genetics, The University of Alabama at Birmingham, Birmingham, Alabama, United States of America; 3 Department of Internal Medicine, The Second Affiliated Hospital of Zhengzhou University, Zhengzhou, China; 4 Human Oncology & Pathogenesis Program, Memorial Sloan-Kettering Cancer Center, New York, New York, United States of America; 5 Macau Institute for Applied Research in Medicine and Health, State Key Laboratory of Quality Research in Chinese Medicine, Macau University of Science and Technology, Avenida Wai Long, Taipa, Macau. China; 6 Department of Biochemistry and Molecular Biology, College of Medicine, University of Florida, Gainesville, Florida, United States of America; University of Wisconsin Madison, UNITED STATES

## Abstract

More than 60% of myeloid dysplasia syndrome (MDS) contains mutations in genes encoding for splicing factors such as SF3B1, U2AF, SRSF2 and ZRSR2. Mutations in SF3B1 are associated with 80% cases of refractory anemia with ring sideroblast (RARS), a subtype of MDS. SF3B1^K700E^ is the most frequently mutated site among mutations on SF3B1. Yet the molecular mechanisms on how mutations of splicing factors lead to defective erythropoiesis are not clear. SF3B1^K700E^ mutant binds to an RNA binding protein, RBM15, stronger than the wild type SF3B1 protein in co-immunoprecipitation assays. In addition, K700E mutant alters the RNA splicing of transcription factors TAL1 and GATA1. Via alternative RNA splicing, a novel short TAL1 transcript variant (TAL1s) is generated. Enhanced interaction between SF3B1 and RBM15 promotes the production of full-length TAL1 (TAL1fl) mRNA, while reduction of RBM15 protein level via PRMT1-mediated degradation pathway changes TAL1s/TAL1fl ratio in favor of TAL1s. TAL1s contains the helix-loop-helix DNA binding domain but not the N terminal region upstream of the DNA binding domain. The TAL1s protein loses its interaction with ETO2, which represses early erythropoiesis. In this vein, overexpression of TAL1s stimulates the transcription of β-hemoglobin in human leukemia K562 cells and promotes erythroid differentiation of human cord blood CD34^+^ cells cultured in erythropoietin-containing medium. Therefore, mutations of SF3B1 may block erythropoiesis via dysregulation of alternative RNA splicing of transcription factor TAL1, and targeting PRMT1 may alleviate the anemic symptoms in MDS patients.

## Introduction

Myeloid dysplasia syndrome (MDS) is a hematopoietic stem cell disease with defective differentiation capability for multiple lineages. Genome-wide sequencing has identified recurrent mutations in splicing factor genes such as splicing factor 3b subunit 1 (SF3B1) and other splicing genes [1, 2]. Mutations of different splicing factors in individual patients are mutually exclusive to each other, indicating non-redundant roles of splicing factors to different types of MDS [2]. The importance of these splicing factor mutations in MDS has been demonstrated by mutation knock-in mouse models [3–6]. More than 85% of refractory anemia with ring sideroblast patients (RARS) contains mutations in SF3B1 gene with SF3B1^K700E^ as the most frequent mutation. SF3B1^K700E/+^ knock-in mice mimic anemia found in human MDS patients [7]. SF3B1 is a component of U2snRNP, which recognizes the branch point sequences in introns for lariat structure formation during RNA splicing [8]. However how SF3B1 mutant is recruited to intronic sequences is not clear. Previously we demonstrated that RBM15 (RNA Binding Motif Protein 15), an RNA-sequence specific binding protein, is responsible for recruiting SF3B1 to certain intronic elements [9]. Thus, we speculate that dysregulation of the interaction between RBM15 and SF3B1 may play critical roles in the pathogenesis of MDS.

RBM15 is involved in normal and malignant hematopoiesis [10]. Previous studies from our lab have shown that RBM15 controls the alternative RNA splicing of key transcription factors such as RUNX1 and GATA1 by directly binding to the intronic regions of their pre-mRNAs to recruit SF3B1 [9]. In this report, we further analyzed the alternative splicing of another transcription factor, T-cell Acute Lymphocytic Leukemia Protein 1 (TAL1) for its role in erythroid differentiation. TAL1 encodes a helix-loop-helix transcription factor, which is required for early hematopoiesis and for the generation of erythrocytes and megakaryocytes in adult hematopoiesis [11]. TAL1 is one of the nine transcription factors critical for the formulation of the transcriptional regulatory network for hematopoiesis [12, 13]. Dysregulation of TAL1 expression has been associated with more than 60% of T cell acute lymphocytic leukemia (T-ALL) [14, 15]. In a subgroup of T-ALL, mutated TAL1 gene can only produce a short form TAL1 transcript variant (TAL1s) from an intragenic promoter [16]. TAL1s is sufficient to induce leukemia in combination with another E-box transcription factor (Lim domain only 1) LMO1. However, whether the intragenic promoter is active in normal myeloid cells remains unknown. Overexpression of the shortest form TAL1 (TAL1s), which does not have N-terminal region upstream of the DNA binding domain, promotes erythroid differentiation of mouse bone marrow cells [17]. Here we report that the TAL1fl can be generated in myeloid cells by RBM15-mediated alternative splicing via recruiting SF3B1, while TAL1s is generated by the attenuated interaction between RBM15-SF3B1. TAL1s activates the transcription of ALAS2 and β-hemoglobin expression, while TAL1fl does not activate β-hemoglobin expression. Overexpression of TAL1s in human CD34^+^ cells promotes erythroid differentiation. At molecular level, we found that TAL1s but not the TAL1fl loses the interaction with ETO2. Transcription corepressor ETO2 interacts with TAL1 and blocks early erythropoiesis [18, 19]. Consistently, we found that SF3B1 mutant blocks efficient erythroid differentiation, presumably by changing the ratio of TAL1s/TAL1fl in favor of TAL1fl.

The RBM15 protein is arginine methylated by protein arginine methyltransferase 1 (PRMT1) and subsequently the methylated RBM15 is degraded via CNOT4 (CCR4-NOT Transcription Complex Subunit 4)-mediated ubiquitylation [9]. Thus, the PRMT1-RBM15 axis modulates alternative RNA splicing via arginine methylation. Using isoform specific primers to perform real-time PCR assays, we have validated that downregulation of RBM15 or upregulation of PRMT1 expression levels promote the production of mRNAs of GATA1s and RUNX1a, and here we showed that TAL1s is generated in the same manner. Thus, arginine methylation by PRMT1 not only blocks megakaryocyte differentiation but also promotes erythrocyte differentiation via fine-tuning the expression levels of different isoforms of TAL1. Taken together, these results suggest that enhancing PRMT1 activity could attenuate the interaction between SF3B1 mutant and RBM15 to stimulate erythroid differentiation via TAL1s.

## Materials and methods

### Purification of cord blood CD34^+^ cells

Human umbilical cord blood was collected from healthy pregnant women with informed consent. After separation with Ficoll gradient, the CD34^+^ cells were purified with anti-CD34 magnetic beads (Miltenyi Biotec, Bergisch Gladbach, Germany) according to manufacturer's instruction and cultured in Iscove’s modified Dulbecco’s medium (IMDM) (GIBCO) supplemented with 20% serum substitutes (BIT) (Stem Cell Technology, Canada) and a cytokine mixture (20ng/ml thrombopoietin (TPO), 20ng/ml interleukin-6, 100ng/ml stem cell factor (SCF), 10 ng/ml Flt3 ligand from Peprotech). The purification achieved 95% pure for CD34^+^ cells based on FACS analysis. To induce MK differentiation, the expanded CD34^+^ cells were cultured in IMDM medium with cytokine mixture (50 ng/ml TPO +10ng/ml SCF). Expression of CD41, CD42 was analyzed by flow cytometry 10 days after TPO stimulation. We infected the CD34^+^ cells grown in basic cytokine mix (20) using lentiviruses expressing TAL1s and TAL1fl proteins respectively. Forty-eight hours after infection, the sorted GFP-positive cells were cultured in IMDM medium with 2U/ ml of erythropoietin (EPO) and 100ng/ml of SCF for erythroid differentiation.

### Virus production and transduction

Lentivirus plasmids expressing cDNAs (containing only the open reading frames) of TAl1fl, TAL1s, SF3B1, SF3B1^K700E^, PRMT1 V2 (we used transcript variant 2 throughout the experiments mentioned here), RBM15 and TAL1 were transfected into 293T cells with helper plasmids for virus production [9]. The transduced leukemia cells and primary CD34^+^ cells were selected with puromycin or by GFP sorting.

Leukemia cell lines MEG-01, K562 were cultured with RPMI medium with 10% fetal bovine serum.

### Flow cytometry analysis

Cells were analyzed by BD Fortessia machine within 1 hour after staining with antibodies. The data were analyzed with BD FACS Diva software and FlowJo software.

### Measure gene expression level by real time polymerase chain reaction (RT-PCR)

RT-PCR was used to measure mRNA expression levels. Total RNA was isolated using the RNeasy Micro Kit (QIAGEN Cat. 74004). Two micrograms of total RNA were reverse transcribed using SuperScript® First-Strand Synthesis System for RT-PCR (Cat. 11904–018, Thermo Scientific). Real-time PCR was performed using SYBR® Green Real-time PCR Master Mix-Plus (Thermo Scientific). The expression level was normalized to GAPDH expression level using ΔΔCt method. The real-time PCR primers are listed below (Table 1). Of note, the set of real-time PCR primers for TAL1s can only detect the endogenous TAL1s mRNA as the forward primer anneals to 5’UTR. In addition, we have used regular PCR to demonstrate the two transcript variants with the two primers: forward: GAAAAAGGGGGAAAGCAAAG, reverse: GGGGAAGGTCTCCTCTTCAC.

**Table 1:** Real-time PCR primers.

TAL1S forward	5'- CTAAATATGCCCCAGCGG GT-3'
TAL1S reverse	5'-CTACAGTAATCTCCATCTC -3'
β-Globin forward	5’-AACTGTGTTCACTAGCAACCTCAA-3’
β-Globin reverse	5’-GAGTGGACAGATCCCCAAAGGA-3’
γ-Globin forward	5’-GATGCCATAAAGCACCTGGATG-3’
γ-Globin reverse	5'-TTGCAGAATAAAGCCTATCCTTGA-3'
ALAS2 forward	5’-ATCTGTGGCCTCAAAGGATG-3’
ALAS2 reverse	5’-CTGCTCAAGCTCCACATGAA-3’
endogenous TAL1fl forward	5'-GGA TGC CTT CCC TAT GTT CA-3’
endogenous TAL1fl reverse	5'-GAT GTG TGG GGA TCA GCT TG-3’

### Co-Immunoprecipitation assay and western blotting assay

We followed protocol we have established for studying protein-protein interactions in blood cells before [20]. Anti-TAL1 (C-21 sc-12984) and anti-ETO2 (G-20, sc-9741) antibodies were purchased from Santa Cruz biotech. Anti-Flag affinity gel is from Biolegend (651501). Anti-RBM15 (10587-1-AP) antibody is from Proteintech. SF3B1 antibody (PAS-19679) is from Fisher Scientific. All the antibodies were diluted a thousand fold for western blotting.

## Results

### Alternative RNA splicing of TAL1 is regulated by the PRMT1-RBM15 axis

**Fig 1:**
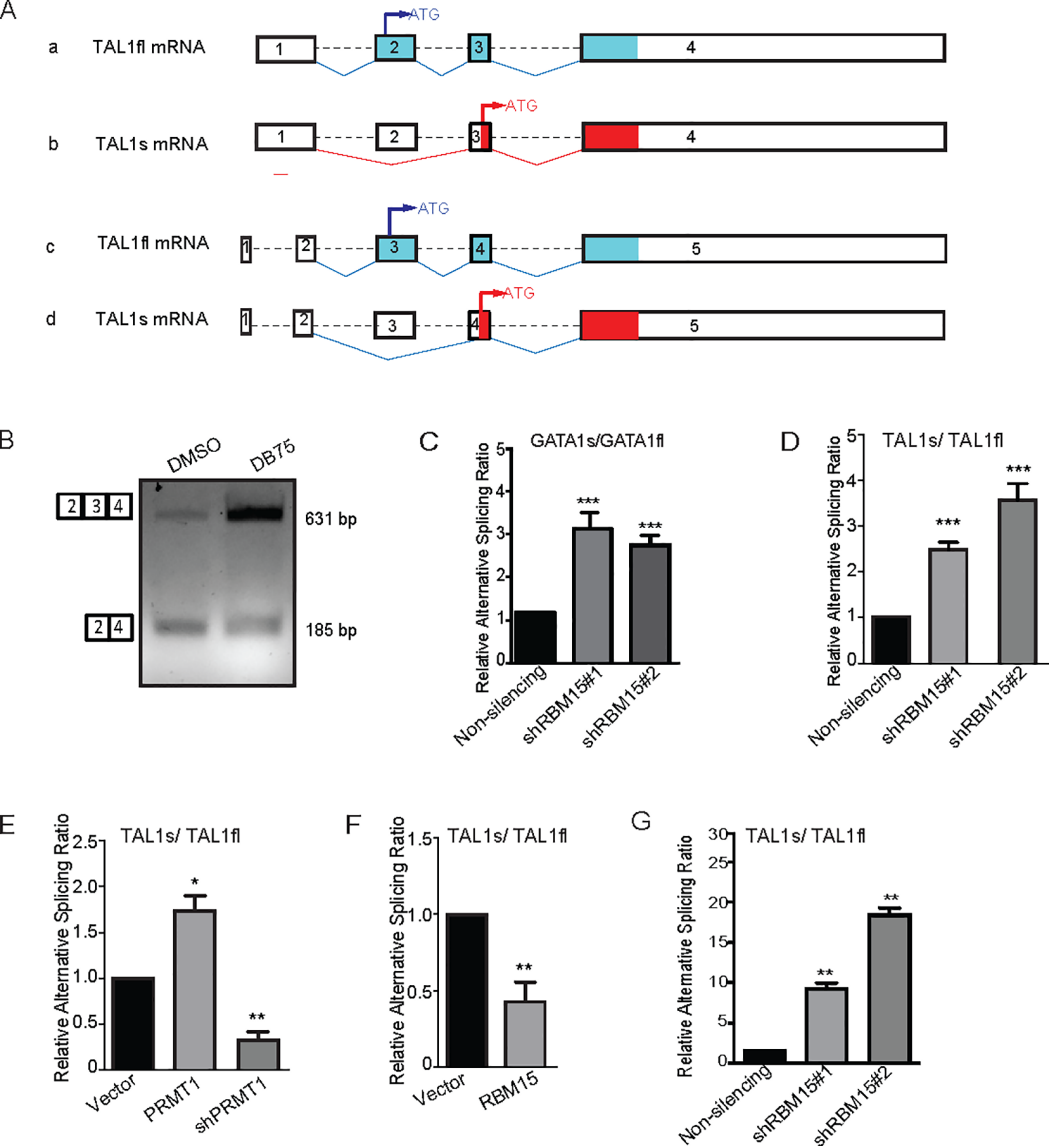
TAL1 encodes transcript variants via alternative RNA splicing. **(A)** Schematic of alternative RNA splicing of TAL1 pre-mRNA to produce TAL1fl and TAL1s via skipping an exon. In the human Ensembl genome database, TAL1 has two transcript variants which have potential to make full-length TAL1 protein. From analysis RNA-seq data from MEG-01 cells, we predict that the exon 2 (transcript a) or exon 3 (transcript c) can be skipped to make two novel variants as shown in b and d which encode TAL1 short form protein (TAL1s). The real-time PCR primers specific for TAL1s cannot distinguish transcript b or d. **(B)** DNA agarose gel to show the two transcript variants as detected by PCR reactions. The forward PCR primer anneals to exon 1 or exon 2 for two long transcript variants and the reverse primer anneals to exon 3 or 4. PRMT1 inhibitor DB75 treated MEG-01 cells were used to compare the amount of full-length TAL1 variants with that of DMSO-treated MEG-01 cells. **(C)** Real-time PCR assays to measure the ratio of GATA1s/GATA1fl in RBM15 knockdown MEG-01 cells. **(D)** The ratio of TAL1s/TAL1fl mRNAs in RBM15 knockdown cells with two different shRNAs against RBM15. Vector expressing non-silencing control shRNA was used as a control. **(E)** Overexpression of PRMT1 and knockdown of PRMT1 in MEG-01 cells changes the ratio of TAL1s/TAL1fl mRNAs. **(F)** Overexpression of RBM15 in MEG-01 cells downregulats the ratio of TAL1s/TAL1fl mRNAs. **(G)** RBM15 knockdown in human CD34^+^ cells grown in basic cytokine mix for two days increases the ratio of TAL1s/TAL1fl mRNAs. Real-time PCR data were presented as means ± standard deviation from three independent experiments. GAPDH mRNA as an internal control. P values were calculated using one-way ANOVA and t test (*p<0.05, **p<0.01, and ***p<0.001).

According to annotation in the Ensembl human genome database, the full-length TAL1 mRNA consists of 4 exons (ENST00000294339.3) or 5 exons (ENST00000371884.6) (Fig 1A). Previously we found that TAL1 is alternatively spliced by RBM15 via direct binding to the intronic regions in TAL1 pre-mRNA [9]. Analysis of RNA-seq data from RBM15 knockdown MEG-01 cells using MISO program indicates that RBM15 promotes the production of full-length TAL1 mRNA, and RBM15 knockdown skips the exon 2 as in the transcript ENST00000294339.3 or exon 3 in the transcript ENST00000371884.6 to produce short isoform as shown on Fig 1A. The short isoform (here we refer to as TAL1s) starts at amino acid 176 of the full length TAL1. To validate the RNA-seq data, we performed regular PCR reactions with cDNA derived from MEG-01 cells with and without DB75 (a PRMT1 inhibitor) treatment (Fig 1B). Inhibition of PRMT1 enzymatic activity by DB75 enhances the production of full-length TAL1 mRNA, which implies that the PRMT1-RBM15 axis is required for the generation of TAL1 full-length mRNA. Given that the primers for regular PCR reactions anneal to common exons in full-length and short form of TALs, we can detect both bands on DNA agarose gel. To further validate how PRMT1-RBM15 axis controls alternative RNA splicing of TAL1, we designed an isoform specific forward primer, which spans the new junction formed by joining exon 2 and exon 4 as in the transcript variant (ENST00000371884.6) to validate in MEG-01 cells the existence of TAL1 short isoform, and a forward primer annealing to exon 3 (ENST00000371884.6) to detect TAL1fl. These isoform specific primers cannot distinguish the two transcripts (ENST00000294339.3 and ENST00000371884.6), therefore we cannot rule out that the TAL1s has two different transcript variants with different 5’UTR regions. Using shRNA to knock down RBM15 increases the ratio in favor of GATA1s as we have reported (Fig 1C) and knockdown of RBM15 favors TAL1s mRNA (Fig 1D). PRMT1 overexpression and RBM15 knockdown increase the ratio of TAL1s/TAL1fl, while PRMT1 knockdown and RBM15 overexpression decrease the ratio of TAL1s/TAL1fl (Fig 1E and 1F). Given that the primer to detect TAL1s spans the junction of exon 2 and exon 4, this primer design excludes the possibilities that an internal promoter downstream of exon 3, which is active in T-ALL cells, is responsible for the generation of TAL1s. These real-time PCR results further validate that RBM15 regulates TAL1 alternative RNA splicing as previously reported. In agreement with data from MEG-01 cells, we further validated that TAL1s/TAL1fl ratio increases in human CD34^+^ cells with RBM15 knockdown (Fig 1G).

### The PRMT1-RBM15 axis regulates the protein concentrations of two TAL1 isoforms

**Fig 2:**
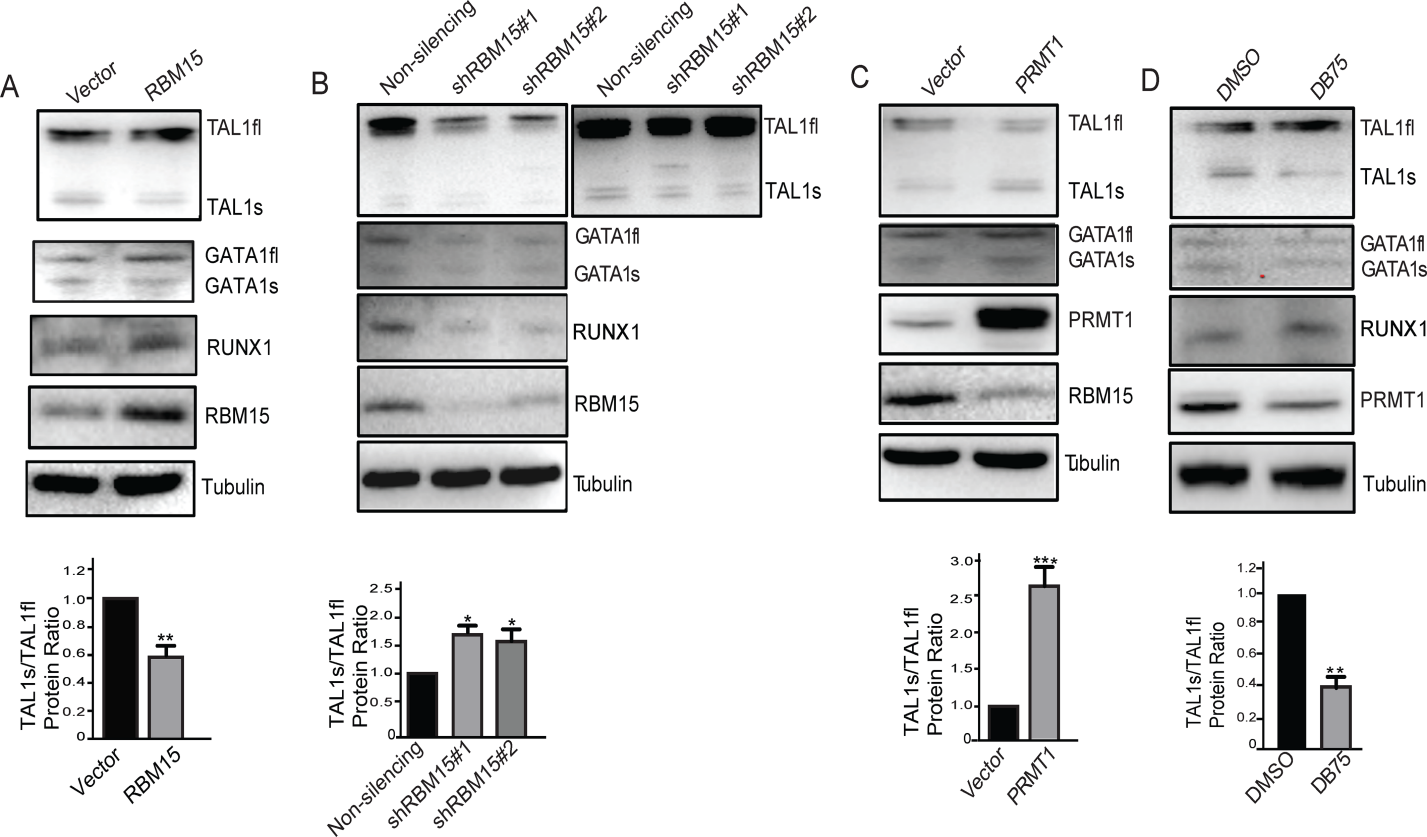
Western blots of TAL1fl and TAL1s protein levels in MEG-01 cells. **(A)** The proteins levels in RBM15 overexpressing MEG-01 cells. **(B**) The protein levels in RBM15 knockdown MEG-01 cells. Lentivirus vector expressing non-silencing shRNA was used as a control. **(C)** The protein levels in PRMT1 overexpressing MEG-01 cells. **(D)** The protein levels of TAL1s and TAL1fl in MEG-01 cells treated with a PRMT1 inhibitor DB75. The ratio of TAL1s and TAL1fl protein concentrations is displayed underneath the corresponding western blots. All western blots were performed at least twice independently. P values were calculated using one-way ANOVA and t test (*<0.05, **<0.01, and ***<0.001).

Changing the ratio of mRNA variants may not have direct impact on TAL1-regulated biology. Only the changes on the protein concentrations have direct impact on biological outcomes. Thus, we wonder how the protein levels of the transcript variants change in response to arginine methylation signals. We detected the decrease of TAL1s protein level when RBM15 is overexpressed in MEG-01 cells as measured by western blotting (Fig 2A). Yet we only observe modest increase of TAL1, GATA1 and RUNX1 full-length protein concentrations in the RBM15 overexpressing cells. Consistently, we observed downregulation of full-length TAL1 protein levels with two different shRNAs against RBM15 (Fig 2B). Give that TAL1s level is relatively low in MEG-01 cells, we also included a darker exposed film to show the expression of TAL1s protein in RBM15 knockdown cells. GATA1 and RUNX1 protein levels are downregulated by knocking down RBM15. Thus, RBM15 is required for producing GATA1, RUNX1 and TAL1 full-length proteins. The anti-RUNX1 N terminal antibody is not good enough to detect RUNX1a. We further tested the TAL1 expression level in PRMT1 overexpressing MEG-01 cells. Overexpression of PRMT1 has modest decrease of the TAL1 full-length protein level but upregulates TAL1s protein level (Fig 2C). When we treated the MEG-01 cells with PRMT1 inhibitor DB75 for 24 hours, TAL1 full-length protein level is increased, TAL1s protein level is decreased (Fig 2D). Quantitation of protein bands shows the protein ratio of TAL1s/TAL1fl is agreeable with mRNA ratio. In aggregates, these western blot data further showed that alteration of PRMT1 and RBM15 dosages directly impact on protein levels of the two different TAL1 isoforms in the opposite directions.

### Overexpression of TAL1s promotes erythroid differentiation

**Fig 3:**
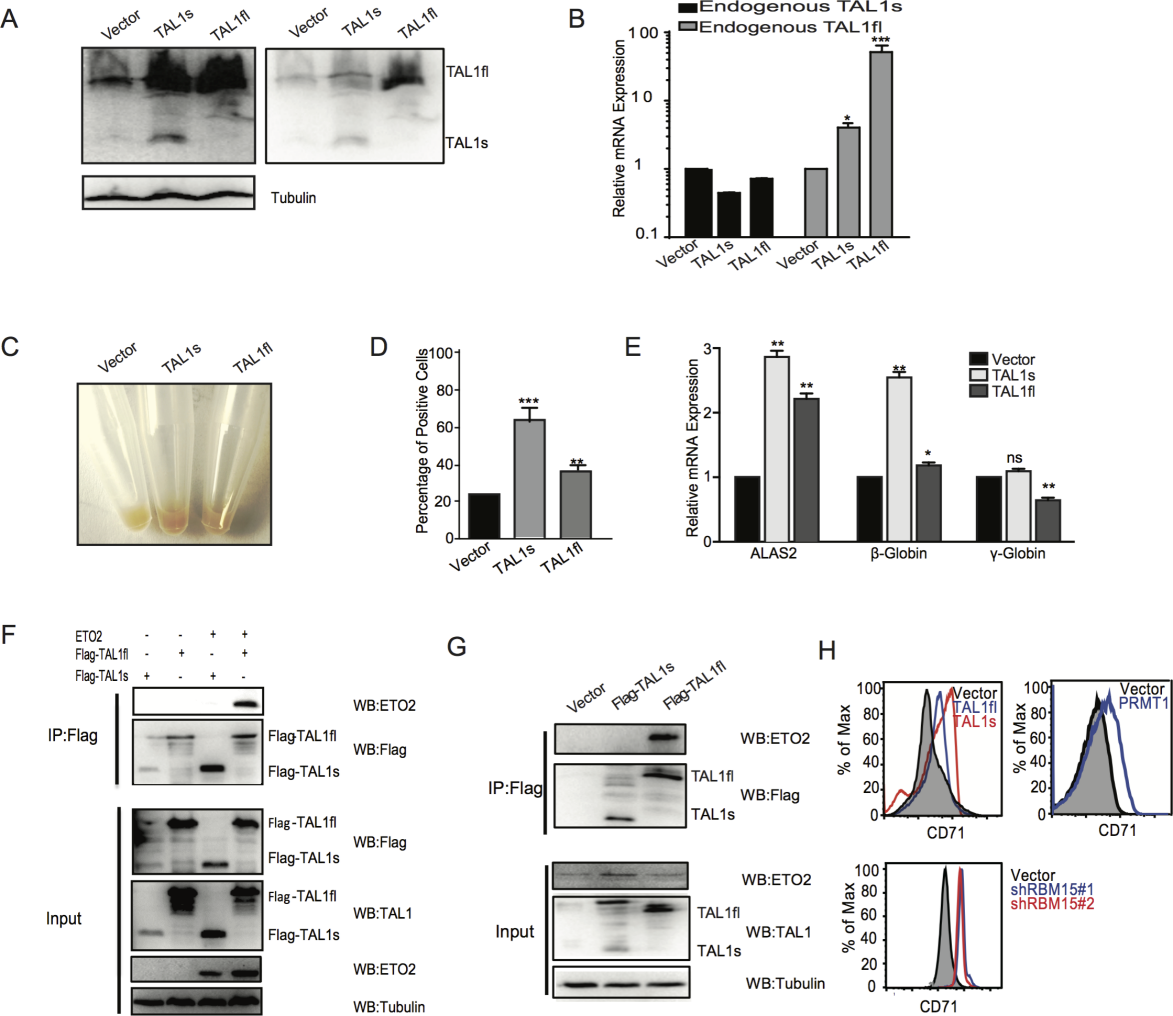
TAL1s promotes erythroid differentiation. **(A)** Western blots to confirm the establishment of K562 cells lines expressing TAL1s and TAL1fl respectively. **(B)** Real-time PCR assays to measure the transcription of endogenous TAL1fl and TAL1s in K562 cells infected with viruses expressing TAL1s and TAL1fl respectively. **(C)** The pellets of the K562 cell lines expressing TAL1s and TAL1fl respectively were compared. **(D)** Benzidine staining of K562 cells lines expressing TAL1s and TAL1fl respectively. The percentage of positively stained K562 cells was calculated. **(E)** The expression levels of ALAS2, β and γ hemoglobin in these established cell lines as listed in panel A. The levels of mRNAs were calculated as mean ± standard deviation from three independent experiments. **(F)** Co-immunoprecipitation assay with Flag antibody to pulldown Flag-tagged TAL1s and Flag-TAL1fl. 293T cells were transfected with plasmids expressing ETO2 and Flag-tagged TAL1 proteins in combination as indicated above the gel. **(G)** Co-immunoprecipitation assay with Flag antibody to immunoprecipitate Flag-tagged TAL1s and Flag-TAL1fl. The co-immunoprecipitated ETO2 was detected by anti-ETO2 antibody. K562 cells were used to make stable cell lines to express Flag-tagged TAL1s and Flag-TAL1fl respectively. **(H)**The percentages of CD71^+^ erythroid cells were shown in histograms. Human CD34^+^ cord blood cells were infected with viruses expressing PRMT1, TAL1s, TAL1fl and shRNAs against RBM15 respectively. After grown in EPO containing medium for 10 days, FACS analysis was performed for CD71 labeled cells. P values were calculated using one-way ANOVA and t test (*<0.05, **<0.01, and ***<0.001).

TAL1s encodes a truncated TAL1 protein with the same amino acid sequence as reported as TAL1D before [17]. Overexpression of TAL1D in mouse bone marrow cells promotes erythroid differentiation more efficiently than the full-length TAL1. Given that TAL1s does not contain N terminal region upstream of the DNA binding domain, TAL1s may function differently as compared to full-length TAL1 protein. We ectopically expressed TAL1s and TAL1fl in K562 cells respectively using a lentiviral vector. As shown in Fig 3A, overexpression of TAL1s enhances the protein expression of endogenous TAL1fl, and intriguingly the TAL1fl overexpressing cells only expresses the TAL1fl without upregulation of TAL1s. We obtained the same results using MEG-01 cells (S1 Fig). In addition, we found that overexpression of TAL1s did not upregulate the endogenous TAL1s mRNA levels, while overexpression of TAL1fl upregulates the transcription of endogenous TAL1fl but not the TAL1s mRNA (Fig 3B). Both the TAL1s and TAL1fl promote the differentiation of K562 cells as the pellets of TAL1fl or TAL1s overexpressing cells turn red. The TAL1s expressing K562 cells are redder (Fig 3C). We stained the K562 cells with benzidine, which stains hemoglobin. K562 cells overexpressing TAL1s have higher percentage of positively stained cells than K562 cells expressing TAL1fl (Fig 3D). Strikingly, the expression of TAL1s promotes K562 cells to produce more β-hemoglobin mRNA than the expression of TAL1fl. On the other hand, TAL1 full-length as well as the short form activates the transcription of ALAS2, which is responsible for heme production (Fig 3E). To further understand the mechanism of TAL1s-mediated erythroipoiesis, we performed immunoprecipitation assays with the two different TAL1 isoforms. ETO2 has been shown to be involved in blocking early erythroid differentiation [18, 19, 21]. Our co-immunoprecipitation assay showed that TAL1s lost its interaction with ETO2 while TAL1fl binds to ETO2 as shown in 293T cells (Fig 3F) as well as in K562 cells (Fig 3G). CD34^+^ cells were transduced with lentivirus expressing TAL1s, TAL1fl, PRMT1 and shRNAs against RBM15 respectively, and were cultured with EPO-containing medium. We found that overexpression of PRMT1 or knockdown of RBM15 promotes the differentiation into erythroid lineage as shown by higher percentage of CD71^+^ cells in FACS analysis. TAL1s also promotes the differentiation into erythroid lineage more efficiently than TAL1fl (Fig 3H), which can be explained by PRMT1-mediated enhancement of TAL1s production. In summary, we demonstrated that the PRMT1-RBM15 axis promotes erythropoiesis partly via upregulation of TAL1s-target genes.

### SF3B1^K700E^ mutant binds to RBM15 more tightly to dysregulate alternative RNA splicing of TAL1

**Fig 4:**
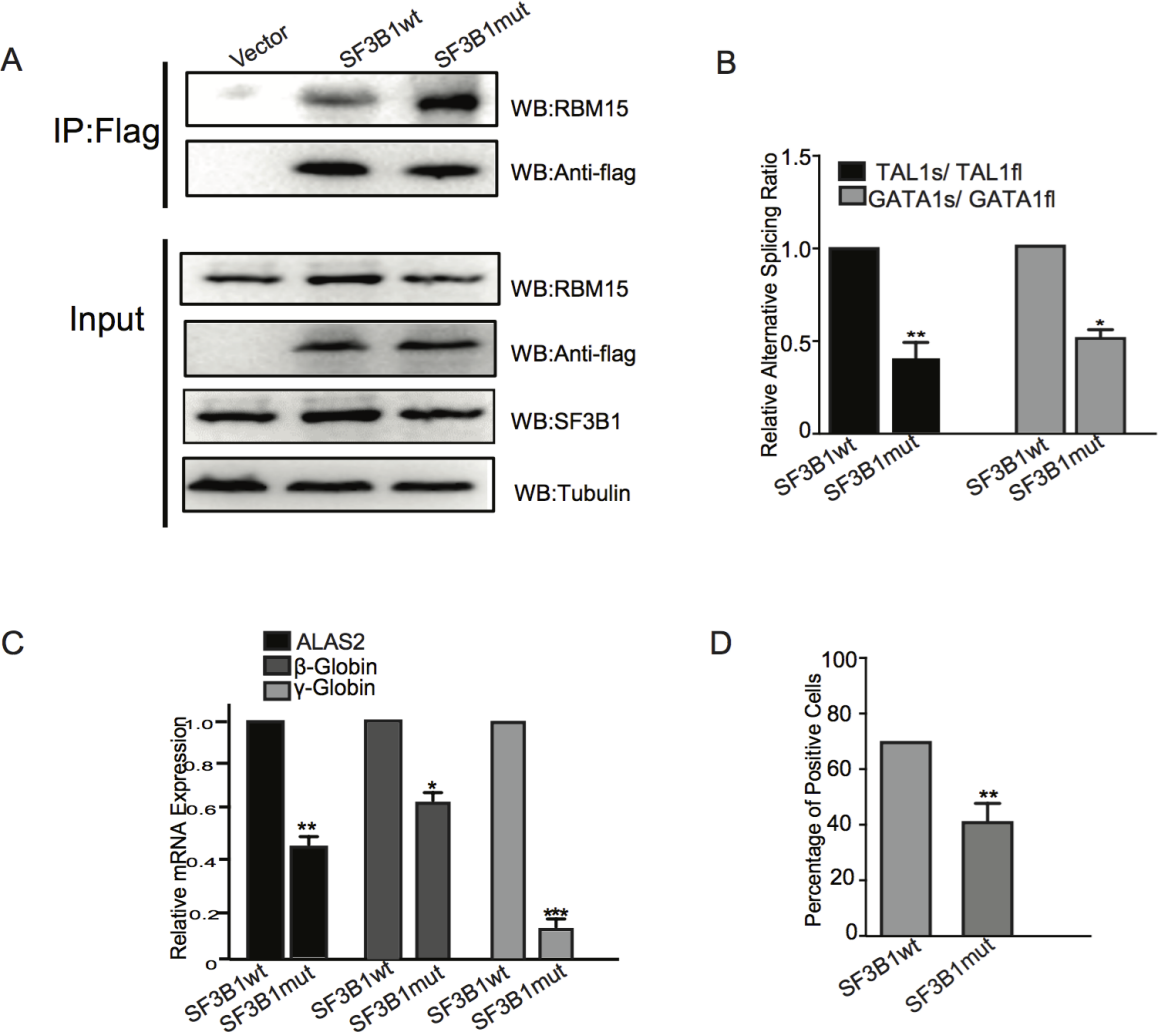
SF3B1 K700E impedes erythroid differentiation via enhancing RBM15-mediated RNA splicing. **(A)** Coimmunoprecipitation assays: Flag tagged SF3B1 wild type and K700E overexpressed in K562 cells were used for immunoprecipitation with Flag antibody. The co-immunoprecipitated RBM15 protein was detected by Western blots with anti-RBM15 antibody. (**B**) Real-time PCR assays to detect the isoforms of GATA1 and TAL1. The ratio of the two isoforms were calculated and compared between wild type and mutant SF3B1 expressing cells. (**C**) The gene expression levels of the erythroid-specific genes ALAS2 and hemoglobin β and γ were measured by real-time PCR assays. (**D**) The benzidine-positive cells were measured among K562 cells overexpressing SF3B1 wild type and mutant respectively. The K562 cell lines were grown in regular medium without EPO.

Previously, we have demonstrated that RBM15 is responsible for recruiting SF3B1 splicing factor to the intronic elements of RUNX1, GATA1, and TAL1 pre-mRNAs. We asked whether RBM15 may still bind to mutated SF3B1. To address this question, we made lentivirus vector to express the K700E mutant ectopically in K562 cells. Ectopic expression of SF3B1 does not increase the total level of SF3B1 protein significantly according to western blots (Fig 4A). Then we immunoprecipitated the SF3B1 mutant as well as the wild type via the Flag tag on SF3B1 N terminals. Compared to wild type SF3B1, the K700E mutant immunoprecipitates more RBM15 protein, indicating that the interaction of RBM15 and the mutant is enhanced (Fig 4A). Thus, it is possible that strengthened interaction between RBM15 and SF3B1^K700E^ may enhance the splicing reaction in favor of TAL1fl and GATA1fl. Using isoform specific primers, we showed that SF3B1 promotes the ratio of TAL1s/TAL1fl in favor of TAL1fl (Fig 4B). Consistently we showed that SF3B1 decrease the ratio of TAL1s/TAL1fl as well as the mRNA levels of ALAS2 and hemoglobin genes (Fig 4C), which are activated by TAL1s (Fig 3E). As a result, SF3B1 mutant expressing K562 cells have less benzidine positive cells (Fig 4D). In summary, our data illustrated that SF3B1^K700E^ in MDS attenuates erythropoiesis via the PRMT1-RBM15 axis, which controls the concentrations of transcript variants of transcription factors such as TAL1 known important for erythropoiesis.

## Discussion

Combinatorial actions of a limited number of transcription factors with different expression levels ultimately determine the distinct paths blood stem/progenitor cells will take in differentiation [22, 23]. Genes encoding for transcription factors produce mRNA isoforms or transcript variants, which may encode proteins that have very distinct and even opposite functions in regulation of hematopoiesis [24]. Transcriptome-wide RNA-seq analysis of different blood lineage cells elucidates the critical roles of alternative RNA splicing of transcription factors such as NFIB for megakaryocyte differentiation [25]. We previously published that a RNA binding protein RBM15 regulates alternative RNA splicing of RUNX1 and GATA1 and promotes megakaryocyte terminal differentiation. In this report, we revealed that a key transcription factor TAL1 important for megakaryocyte-erythroid progenitor differentiation is alternatively spliced by the PRMT1-RBM15 axis. Analysis of RNA-seq data we published previously (Zhang et al 2015) showed that the pre-mRNA of transcription factor TAL1 is alternatively spliced to yield a short transcript variant (TAL1s). Based on the position of the first ATG in the TAL1s mRNA, the short form TAL1 protein starts at 176a.a. of the full-length TAL1. TAL1s is of the same length as predicted previously to be produced by using alternative protein translation or using internal transcription start site. Given that protein translation often starts at the first ATG via ribosomal scanning mechanism [26], the TAL1fl mRNA will produce full-length TAL1 protein in most cases. However, under stress conditions Dr. Leutz’s lab demonstrated that a uORF (upstream ORF in 5’UTR) regulates the protein translation of TAL1s using internal ATG site of the full-length TAL1 mRNA [17]. Given that the internal transcription start site is downstream of exon 3 [27] and that the primer we designed to detect the short isoform spanning the junction of exon 2 and 4, we rule out the possibility that TAL1s we detected is transcribed via the intragenic promoter. Up to now, the activity of intragenic promoter is only reported in T leukemia cells [27]. In summary, TAL1s can be generated via PRMT1-mediated methylation signals i.e. alternative RNA splicing regulation as well as via assembly different protein translational machineries on the different AUG start sites in full-length TAL1 mRNA in response to PKR phosphorylation signals.

To understand the functions of TAL1s in hematopoiesis, we ectopically expressed TAL1s. TAL1s stimulates K562 cells to make more hemoglobin, which is consistent with previous data from Dr. Leutz’s lab that TAL1s promotes erythropoiesis using mouse bone marrow cells. TAL1 has been demonstrated to be required for the looping of LCR to specific hemoglobin promoters [28]. Given that TAL1s not the TAL1fl activates the beta hemoglobin, the balance between TAL1s and TAL1fl determines the transcriptional regulation of ß hemaglobin. More future study is needed to work out the mechanistic detail of TAL1s-mediated erythroid differentiation. TAL1 target genes have been extensively studied via ChIP-seq analysis. However, the antibodies for TAL1 c-terminal region cannot distinguish the isoforms. A TAL1s specific transcriptional regulatory complex for erythropoiesis, which bypasses the repression of ETO2, should be characterized in the future. Given that TAL1s exists in zebra fish albeit with different length [29], most likely many functions of TAL1s are evolutionally conserved. In zebra fish TAL1s protein is very unstable, although the mRNA levels of TAL1s and TAL1fl are similar. From Fig 1B, we also observed similar mRNA levels of the two TAL1 transcript variants. Consistent with zebrafish data, we also found that TAL1s in human cells is very unstable presumably via ubiquitylation as implied by our unpublished data. Elucidating the unique functions of TAL1s may lay fundamentals for understanding normal hematopoiesis, T cell acute leukemia as well as anemia associated with many types of hematological malignancies such as MDS.

In this report, we further demonstrated that alternative RNA splicing of TAL1 and GATA1 is regulated by mutated SF3B1 as the ratio of the two isoforms changes to favor the full length. Given that RBM15 binds to RNA sequence specifically, defining RBM15 binding sequence with single nucleotide resolution assays such as PAR-CLIP will further help us to define SF3B1 mutant target genes in the future. This contribute may explain the mechanism on how SF3B1 mutant recognize putative branch sites [30]. Spliceosome assembly inhibitors have been tested to treat MDS [31]. More research on how specific spliceosomes are assembled by RNA-sequence specific binding proteins such as RBM15 will lead to design more mutation specific splicing inhibitors in the future. The role of arginine methylation as RNA splicing regulators is being increasingly appreciated now [32–39]. Using small molecules to modulate the enzymatic activities of protein arginine methyltransferases may be a feasible approach to treat MDS in the future.

## Conclusions

The SF3B1 splicing factor mutant K700E blocks erythroid differentiation via dysregulation of TAL1 transcription factor alternative RNA splicing. The expression of short transcript variant of TAL1 is critical for production of hemoglobin and efficient erythropoiesis. By enhancing the interaction between RBM15 and SF3B1^K700E^, the relative concentration of TAL1s is reduced.

## Supporting information

S1 Fig Western blots to confirm the establishment of MEG01 cells lines expressing TAL1s and TAL1fl respectively. Cell lysates from MEG01 cells expressing TAL1s and TAL1fl were loaded onto SDS-PAGE gel. The protein bands corresponding to TAL1s and TAL1fl were detected by western blotting with antibody against TAL1. Anti-tubulin western blot was used as a loading control.
(TIFF)
