# Supplementary figures and images for: Splicing factor SF3B1K700E mutant dysregulates erythroid differentiation via aberrant alternative splicing of transcription factor TAL1
Source: PLoS One. 2017 May 18;12(5):e0175523. doi: 10.1371/journal.pone.0175523 (PMC5436638; doi:10.1371/journal.pone.0175523)

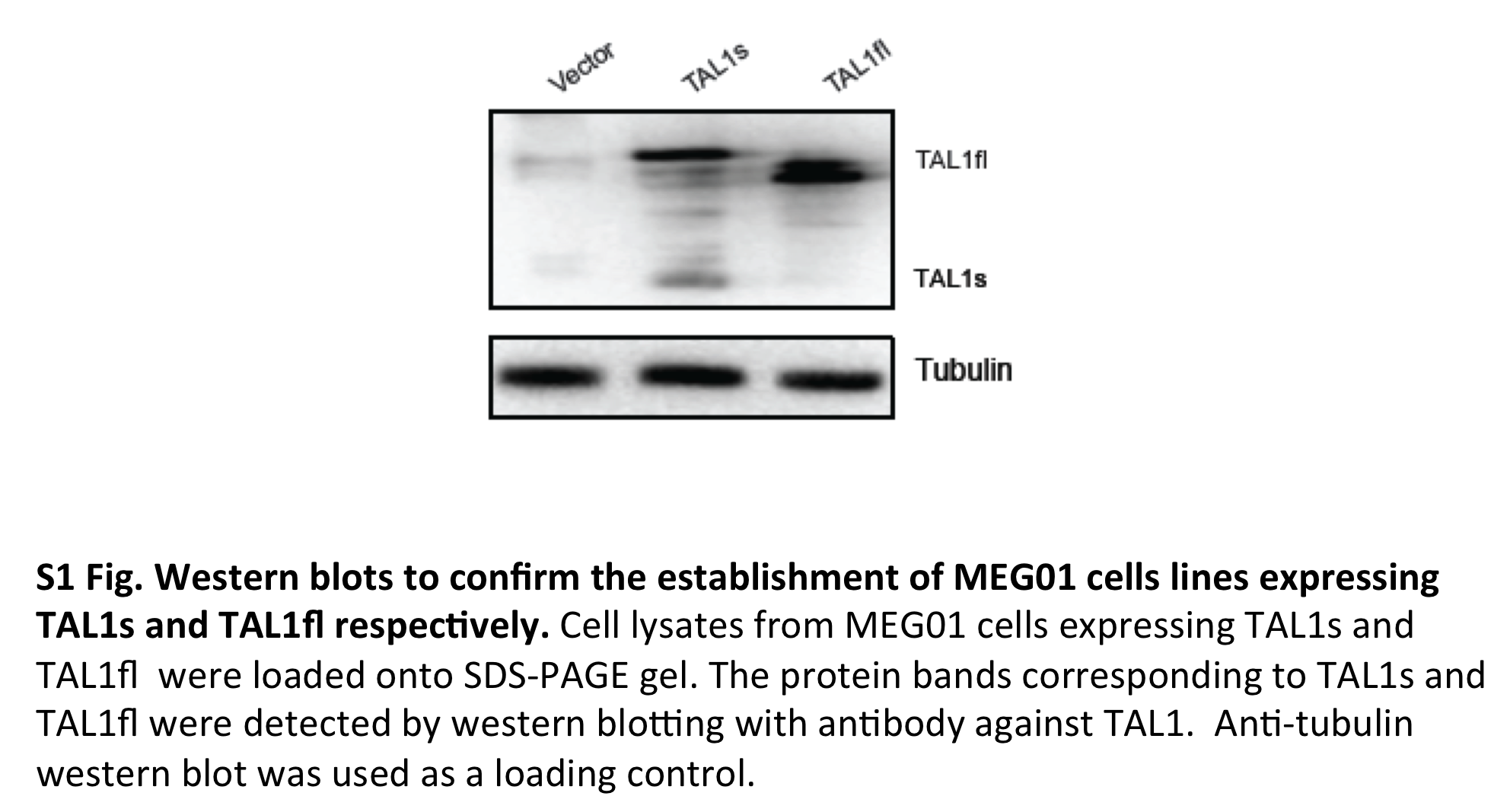

Supplement: S1 Fig — Cell lysates from MEG01 cells expressing TAL1s and TAL1fl were loaded onto SDS-PAGE gel. The protein bands corresponding to TAL1s and TAL1fl were detected by western blotting with antibody against TAL1. Anti-tubulin western blot was used as a loading control. (TIFF) [file pone.0175523.s001.tiff]
